# Death from 1918 pandemic influenza during the First World War: a perspective from personal and anecdotal evidence

**DOI:** 10.1111/irv.12267

**Published:** 2014-06-27

**Authors:** Peter C Wever, Leo van Bergen

**Affiliations:** a Department of Medical Microbiology and Infection Control, Jeroen Bosch Hospital′s-Hertogenbosch, The Netherlands; b Military Medicine Historical Research SocietyThe Netherlands; c KITLV (Royal Netherlands Institute of South East Asian and Caribbean Studies)Leiden, The Netherlands

**Keywords:** 1918 Pandemic influenza, First World War, mortality, risk factors, secondary bacterial pneumonia, Spanish influenza

## Abstract

The Meuse-Argonne offensive, a decisive battle during the First World War, is the largest frontline commitment in American military history involving 1·2 million U.S. troops. With over 26 000 deaths among American soldiers, the offensive is considered “America's deadliest battle”. The Meuse-Argonne offensive coincided with the highly fatal second wave of the influenza pandemic in 1918. In Europe and in U.S. Army training camps, 1918 pandemic influenza killed around 45 000 American soldiers making it questionable which battle should be regarded “America's deadliest”. The origin of the influenza pandemic has been inextricably linked with the men who occupied the military camps and trenches during the First World War. The disease had a profound impact, both for the military apparatus and for the individual soldier. It struck all the armies and might have claimed toward 100 000 fatalities among soldiers overall during the conflict while rendering millions ineffective. Yet, it remains unclear whether 1918 pandemic influenza had an impact on the course of the First World War. Still, even until this day, virological and bacteriological analysis of preserved archived remains of soldiers that succumbed to 1918 pandemic influenza has important implications for preparedness for future pandemics. These aspects are reviewed here in a context of citations, images, and documents illustrating the tragic events of 1918.

It stalked into camp when the day was damp

*And chilly and cold*.

It crept by the guards

And murdered my pards

With a hand that was clammy and bony and bold;

And its breath was icy and mouldy and dank,

And it killed so speedy

And gloatingly greedy

*That it took away men from each company rank*.

From *The Flu* by Private Josh Lee, 1919

## Introduction

**Figure 1:**
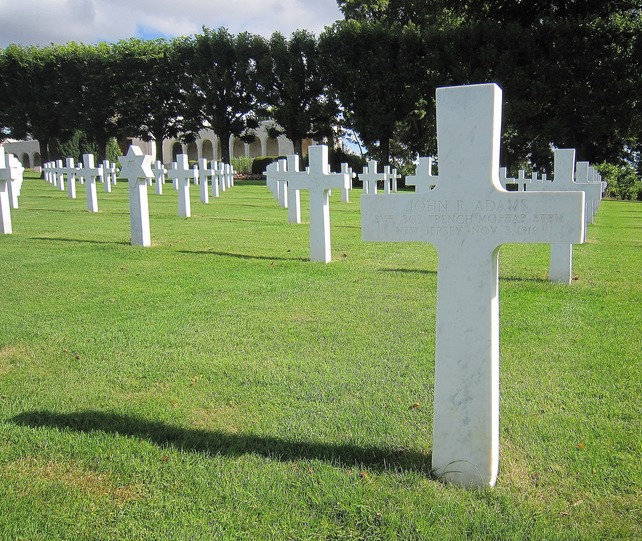
Headstone of U.S. Army private John R. Adams, who is interred in the Meuse-Argonne American Cemetery (Plot G, Row 36, Grave 32) after succumbing to influenza and pneumonia at the age of 26 on November 3, 1918 during the Meuse-Argonne offensive (photograph by first author; courtesy of the American Battle Monuments Commission).

The Meuse-Argonne offensive was a decisive battle during the First World War (WWI) between allied American and French forces and German troops in Northern France. Over the course of 47 days, the allied campaign, which began on September 26, 1918, contributed to the Armistice of November 11, 1918. Involving 1·2 million U.S. troops, the Meuse-Argonne battle is the largest frontline commitment in American military history. With 26 277 deaths among the often inexperienced American soldiers, it is also considered “America's deadliest battle”.^1^ Consequently, the largest number of U.S. military casualties in Europe, 14 246 soldiers, rest within the Meuse-Argonne American Cemetery at Romagne-sous-Montfaucon.^2^ It has been stated, however, that more Americans were buried in France because of 1918 pandemic influenza (Spanish flu) than of enemy fire.^3^ The Meuse-Argonne offensive coincided with the second wave of the influenza pandemic, which ran its deadly course on the Western Front in about eight weeks, from roughly September 15 to November 15, 1918.^4^,^5^ Private John R. Adams is interred in the Meuse-Argonne American Cemetery after succumbing to influenza and pneumonia on November 3, 1918, while serving with the 78th Infantry Division (Figure1).^6^ His death is among the 15 849 caused by 1918 pandemic influenza in the American Expeditionary Forces (AEF) operating in Europe.^7^ As influenza killed almost 30 000 men in U.S. Army training camps before they could even embark to France,^4^,^5^ the overall death toll from 1918 pandemic influenza in the U.S. Army by far exceeds the number of deaths resulting from the Meuse-Argonne offensive, making it questionable which battle should be regarded “America's deadliest”.

Pandemic influenza struck all the armies, but the highest morbidity rate was found among the Americans as the disease sickened 26% of the U.S. Army, over one million men.^3^–^5^ In comparison, the German Army recorded over 700 000 cases of influenza, while the British Expeditionary Forces (BEF) listed 313 000 cases in 1918 in France.^4^

Here, in a context of personal and anecdotal evidence, we set out to review different aspects of mortality from 1918 pandemic influenza among soldiers during WWI, some of which bear significance to preparedness for future pandemics.

## The origin of 1918 pandemic influenza in relation to WWI

A notable characteristic of 1918 pandemic influenza is that it occurred in waves of varying lethality. The first pandemic wave, which took place in the spring of 1918, was relatively mild and caused few deaths. After a period of calm in the beginning of the summer, the virus reemerged in an extremely virulent fashion and caused tens of millions of deaths throughout the world during the second wave in the 1918 autumn months. A third wave, also responsible for considerable mortality, occurred during the initial months of 1919, while a final fourth wave, although not always acknowledged as such, spread during the first months of 1920.^8^,^9^

**Figure 2:**
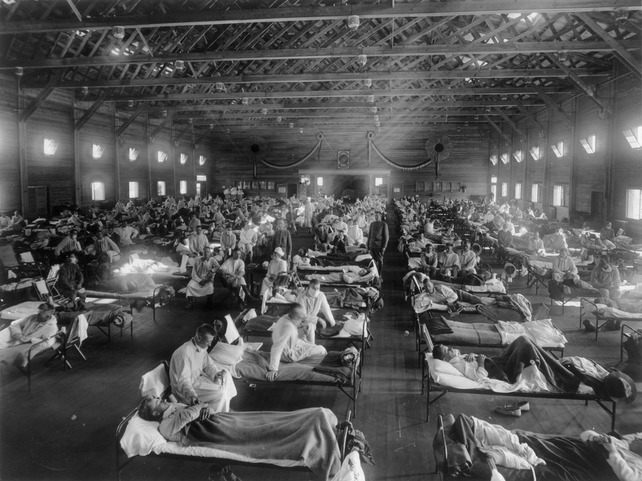
Emergency hospital at Camp Funston, a U.S. Army training camp in Kansas, where the first outbreak generally considered caused by 1918 pandemic influenza occurred (photographer unknown; courtesy of Otis Historical Archives, National Museum of Health and Medicine, Silver Spring, MD, USA).

Although it is problematic to assign a specific date to the beginning of the pandemic, it is clear that its origin was inextricably linked with the millions of men occupying military camps and trenches during WWI. It has been suggested that the beginning of the pandemic occurred in a British military base at Étaples, at the coast of Northern France. The base was crowded with soldiers, was situated near sea marshes with abundant migratory birds, had many farms nearby with pigs, ducks, and geese reserved as food for soldiers, and served as storage of mutagenic war gasses. These conditions might have contributed to an outbreak of acute respiratory infection between December 1916 and March 1917 which clinically resembled 1918 pandemic influenza. The origin of the pandemic has also been traced to Indochinese soldiers from the old Annam kingdom (Vietnam, Laos, Cambodia) fighting in France between 1916 and 1918. Among these soldiers, several epidemics of acute respiratory infections were noted, referred to as Annamite pneumonia.^9^,^10^ Yet, the first outbreak generally considered caused by 1918 pandemic influenza occurred at Camp Funston, a U.S. Army training camp in Kansas.^9^,^11^ In the beginning of March 1918, Chinese contract workers at Camp Funston presented with influenza.^9^ Subsequently, the disease spread across the camp requiring hospitalization of over 1100 soldiers within three weeks besides thousands more receiving treatment at infirmaries around the camp.^11^ Between early March and the summer, five consecutive outbreaks occurred in the camp, coinciding with the arrival of large numbers of new recruits (Figure2).^12^ From Camp Funston on, influenza jumped to other U.S. Army training camps and travelled to Europe aboard troop ships before it subsided in the summer of 1918.^9^,^11^ In all, 11·8% (143 986) of over 1·2 million men in U.S. Army training camps were hospitalized for respiratory illness in March–May 1918, although death rates from respiratory illness showed only a limited increase in that period.^12^

On the Western Front, the first wave impacted both sides of the conflict. In France, influenza appeared in the BEF in April 1918. In the First Army, the total number of admissions to casualty clearing stations for influenza between 18 May and 2 July was 36 473, although a low case mortality rate illustrated the mild character of the first wave. In the Second Army, the highest number of cases admitted to casualty clearing stations for one day was reached on 25 June with 683 admissions.^13^ Correspondingly, the French Army was evacuating 1500–2000 cases of influenza to the rear of the front per day in May 1918.^4^ In his fictionalized autobiography, Siegfried Sassoon, one of the leading British war poets, described the impact of the disease, despite its mild character, on military operations: “The influenza epidemic defied all operation orders of the Divisional staff, and during the latter part of June more than half the men in our brigade were too ill to leave their billets.”^14^ Likewise, German General Erich Ludendorff, joint commander of the German Army, acknowledged the suffering of his army: “Influenza was rampant […]. It was a grievous business having to listen every morning to the chiefs of staffs' recital of the number of influenza cases, and their complaints about the weakness of their troops if the English attacked again.”^15^ Eventually, the first wave subsided in the beginning of the summer of 1918,^9^ although, as General Ludendorff acknowledged, “it often left a greater weakness in its wake than the doctors realized”.^15^

## The second wave

The highly fatal second wave of 1918 pandemic influenza spread globally from September to November 1918 and was responsible for most of its tens of millions of deaths.^8^ It emerged earlier among military forces in Europe than in the USA. Already on 9 August, Colonel Jefferson Kean, Deputy Chief Surgeon of the AEF, documented in his diary its development in France: “Small outbreaks of ‘three-day-fever’ again appear […]; character becomes more virulent,” adding on 17 August that “Influenza [is] increasing and becoming more fatal.” On 18 September, he noted a “Sudden and serious increase in influenza-pneumonia”, which was also observed in the French Army and among civilians. The situation was described as very serious on 6 October, with “Influenza and pneumonia […] increased by thousands of cases. Case mortality of pneumonia, 32 percent,” even further increasing to 45·3% in the week of 11 October. In that week, during the height of the Meuse-Argonne offensive, the highest number of deaths from influenza in the AEF was reached with 1451 reported fatalities.^4^ Even General Ludendorff noted on 17 October that “the fighting power of the Entente [Allies] has not been up to its previous level […] the Americans are suffering severely from influenza”. The influenza pandemic and the Meuse-Argonne offensive stressed the entire medical system, as, by 23 October, there were 20 000 more patients than normal bed capacity in the AEF.^7^ The number of new influenza cases in the AEF declined soon after, however, as noted by Colonel Kean on 26 October.^4^

The second wave likewise hit hard on other armies in Europe. The German Army reportedly lost the lives of 14 000 soldiers to influenza,^3^ although, in all armies, military censorship might have minimized mortality figures. Deaths among non-civilian males allocated to influenza during 1918 in England and Wales numbered 7591.^16^ Captain Geoffrey Keynes of the Royal Army Medical Corps (RAMC) would never forget the sight of the mortuary tents at Bohain, France: “There were rows of corpses, absolutely *rows* of them, hundreds of them, dying from something quite different. It was a ghastly sight, to see them lying there dead of something I didn't have the treatment for.”^17^ The 1945 Nobel Prize winner Alexander Fleming, stationed in Boulogne-sur-Mere, France, as RAMC officer during WWI, found himself carrying corpses of influenza deaths to the improvised cemetery after the orderlies had gone sick.^18^ Due to 1918 pandemic influenza, the number of British medical officers that died from disease was 1·75 times higher in 1918 compared to 1917 with nearly half of the 1918 deaths occurring in October through December.^19^ Even on November 11, 1918, the day of the Armistice, celebrations were tempered by the impact of influenza as recorded by Sister Catherine Macfie at casualty clearing station no. 11 in France: “We moved up to St André after the army went into Lille, and almost immediately we started taking in wounded and many […] who had Spanish influenza as well. […] The boys were coming in with colds and a headache and they were dead within two or three days. Great big handsome fellows, healthy men, just came in and died. There was no rejoicing in Lille the night of the Armistice.”^17^

**Figure 3:**
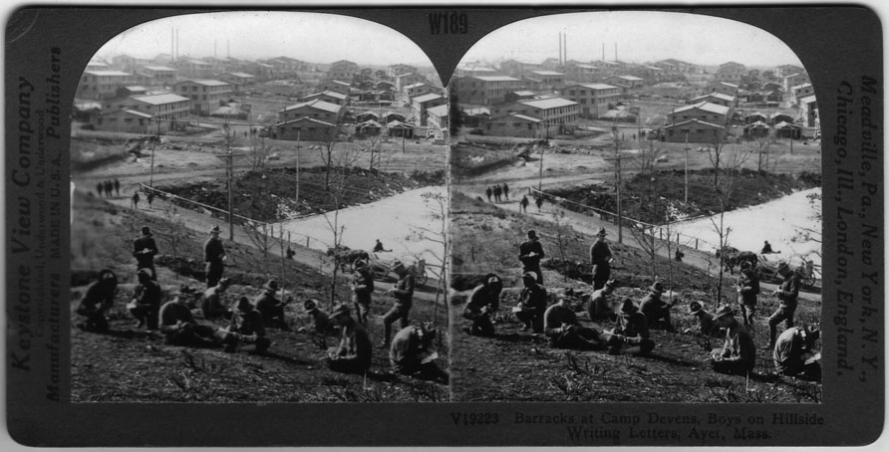
First World War era stereocard view of the barracks at Camp Devens in Massachusetts, which was the first U.S. Army training camp hit by the deadly second wave of 1918 pandemic influenza with more than 14 000 cases and 757 deaths (collection of first author).

**Figure 4:**
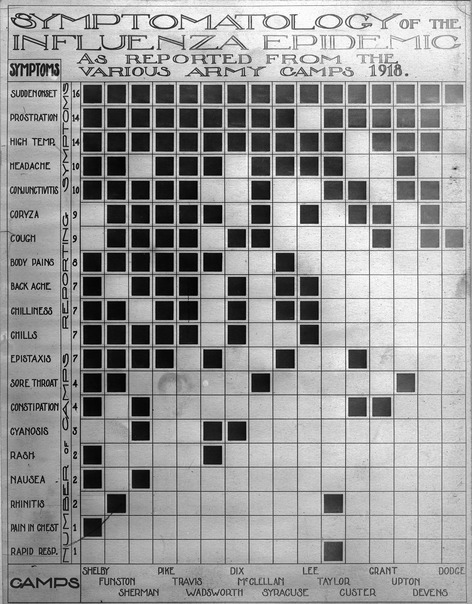
Chart illustrating the symptomatology of 1918 pandemic influenza. Sudden onset, prostration, high temperature, headache, and conjunctivitis were the five symptoms reported most often from 16 U.S. Army training camps in 1918 (Reeve Photograph Collection; courtesy of Otis Historical Archives, National Museum of Health and Medicine, Silver Spring, MD, USA).

In U.S. Army training camps, the second wave emerged on 8 September, when it arrived at Camp Devens, just outside of Boston, Massachusetts,^5^ where more than 100 000 soldiers of the 12th and 76th Infantry Divisions have been trained (Figure3).^20^ Within 10 days, the base hospital and regimental infirmaries were overwhelmed with sick soldiers.^5^ A letter written by a soldier from Supply Company, 74th Infantry Regiment, 12th Division on September 24, 1918 (collection of first author) illustrates the situation in Camp Devens: “Maybe, it was a good thing that I did not come [home], as I probably would have given you this miserable disease that we have here. […] for the last week I have had most everything in that line there ever was, but just now I am better. Have not had too [*sic*] go too [*sic*] the hospital though I have been sick enough too [*sic*] be there, but anyones chances are pretty slim, that goes there so I keep quiet about my being sick and done my own doctoring and think I am better off than most of them. The doctors say that they have got it checked now. I hope so, as every day there has been an average of about twenty deaths here a day, some toll for a camp.” By the end of September, over 14 000 cases of influenza had been noted at Camp Devens, approximately one quarter of its population, resulting in 757 deaths and a case-fatality rate exceeding 5%.^4^ Before a travel ban was imposed, a contingent of replacement troops departed from Camp Devens to Camp Upton, Long Island, New York, where influenza emerged on 13 September.^5^ Soon, other U.S. Army training camps followed as influenza swept from the northeastern coast south and west, following wartime transportation routes.^4^ During the second wave, 27·5% (437 224) of over 1·5 million men in U.S. Army training camps were hospitalized for respiratory illness, with a case-fatality rate that peaked at 5·1% in September,^12^ while in the week of 4 October the highest number of deaths from influenza was reached with 6160 fatalities (Figure4).^4^

## Virology

In 1918, there was neither the knowledge nor the technological tools to identify viruses and their existence could only be theorized. Many scientists mistakenly believed that Pfeiffer's bacillus, currently known as *Haemophilus influenzae*, caused influenza, as it was often found in patients' bodies. Influenza viruses would not be isolated until 1933 and during the pandemic, its cause remained a mystery.^4^ In 1999, Reid *et al*. at the Armed Forces Institute of Pathology published the complete sequence of the hemagglutinin gene of the 1918 pandemic influenza virus, while the sequence of the neuraminidase gene was published the year thereafter. Influenza RNA had been isolated among others from formalin-fixed, paraffin-embedded lung tissues obtained in 1918 during the autopsy of two soldiers, aged 21 and 30, that both had succumbed to 1918 pandemic influenza on 26 September at Fort Jackson, South Carolina, and Camp Upton, respectively. Molecular analysis revealed the virus to belong to the influenza A (H1N1) subtype.^21^,^22^ Using the virus reverse genetics approach, the obtained viral RNA sequences subsequently permitted reconstruction of the complete 1918 pandemic influenza virus.^23^

The pandemic influenza viruses of 1957, 1968, and 2009 descended, via different pathways, from the 1918 pandemic influenza virus. The 2009 influenza A (H1N1) virus (Mexican flu or swine flu) and 1918 pandemic influenza virus express an antigenically similar hemagglutinin which has apparently been transmitted from humans to pigs in or about 1918 to reappear in humans in 2009 as a swine H1 hemagglutinin gene. Consequently, extensive cross-protection was demonstrated between these two pandemic viruses in experimental animals. Furthermore, neutralizing antibodies could still be recovered from elderly survivors 90 years after exposure to 1918 pandemic influenza. Such observations provided rationale for targeting initially limited 2009 influenza A (H1N1) vaccines to younger persons instead of the traditional risk group of elderly.^24^

## Secondary bacterial pneumonia

On September 29, 1918, a U.S. Army physician described the rapid clinical course of fatal influenza at Fort Devens. “These men start with what appears to be an ordinary attack of LaGrippe or Influenza, and when brought to the Hosp. they very rapidly develop the most viscious [*sic*] type of Pneumonia that has ever been seen. Two hours after admission they have the Mahogony spots over the cheek bones, and a few hours later you can begin to see the Cyanosis extending from their ears and spreading all over the face, until it is hard to distinguish the colored men from the white. It is only a matter of a few hours then until death comes, and it is simply a struggle for air until they suffocate. It is horrible. One can stand it to see one, two or twenty men die, but to see these poor devils dropping out like flies sort of gets on your nerves. We have been averaging about 100 deaths per day, and still keeping it up. There is no doubt in my mind that there is a new mixed infection here, but what I don't know.”^25^ This just assumption about the microbiological aspects of fatal 1918 pandemic influenza cases from an otherwise unknown army physician preceded renewed interest in the matter by precisely 90 years.

**Figure 5:**
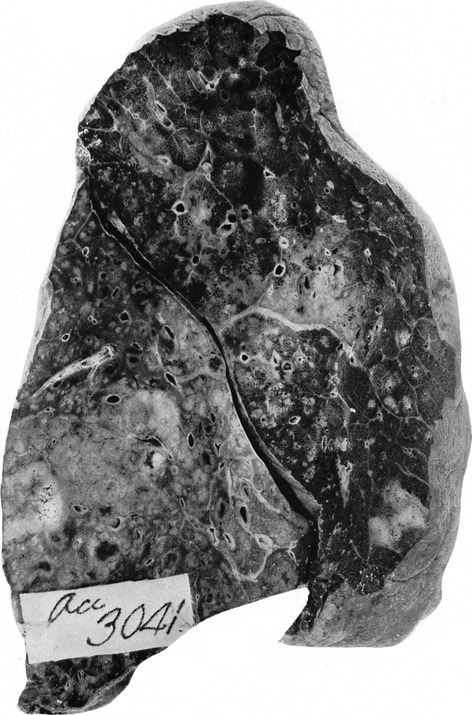
Lung specimen obtained during autopsy of an unknown individual who died of influenza-related pneumonia (accession number 3041, Army Medical Museum). Peribronchiolar consolidations were observed in both lobes extending out from thickened interstitial tissue at about the middle of the upper lobe. Culture revealed *Streptococcus hemolyticus*, currently referred to as β-hemolytic streptococci (photo reprinted with permission from the U.S. Army Medical Department Center of History and Heritage, Research Collection).

In 2008, consistent with epidemiologic and clinical characteristics of the pandemic, expert opinion, and knowledge regarding pathophysiologic effects of influenza viruses and their interactions with respiratory bacteria, Brundage and Shanks introduced the sequential-infection hypothesis, which stated that infections with 1918 pandemic influenza generally caused self-limiting, rarely fatal, illnesses that subsequently enabled colonizing strains of bacteria to produce highly lethal pneumonias.^26^ Concurrently, Morens *et al*. reported results of the examination of recut sections from lung tissues obtained during autopsies from 58 influenza fatalities in 1918–1919 at various U.S. Army training camps, archived at the Armed Forces Institute of Pathology (Figure5). In virtually all cases, compelling histologic evidence of severe acute bacterial pneumonia was revealed. The observed histopathologic spectrum ranged from lobar consolidation with pulmonary infiltration by neutrophils in pneumococcal pneumonia, a bronchopneumonic pattern, edema, and pleural effusions in streptococcal pneumonia and occasionally pneumococcal pneumonia, and multiple small abscesses with marked neutrophilic infiltration in airways and alveoli in staphylococcal pneumonia. Bacteria were commonly observed, often in massive numbers. Correspondingly, bacteriologic and histopathologic results from previously published autopsy series also clearly and consistently implicated secondary bacterial pneumonia caused by common upper respiratory tract bacteria in most 1918 pandemic influenza fatalities. Bacterial culture results additionally implicated *H. influenzae* to appear early in symptomatology in association with diffuse bronchitis and/or bronchiolitis, sometimes infiltrating the bronchiolar submucosa, after which it was typically replaced by other secondary microorganisms.^27^ In 2009, Chien *et al*. reported that bacteria had been recovered in only a few antemortem blood cultures of (predominantly military) patients who had 1918 pandemic influenza without pneumonia (mean <1%), but were commonly isolated from blood cultures of patients with influenza-associated pneumonia (16%), particularly of those who died (40%). *Streptococcus pneumoniae* comprised by far the majority of positive cultures.^28^ Also in 2009, Klugman *et al*. showed that time from onset of illness to death due to influenza-related pneumonia in 1918 among soldiers was comparable with time to death due to pneumococcal pneumonia in the pre-antibiotic 1920s and 1930s. Although similar times to death do not prove a specific bacterial etiology, this observation provided additional evidence that influenza-related pneumonia deaths due to 1918 pandemic influenza were largely due to *S. pneumoniae*.^29^ In the wake of these observations, it has been stated that pandemic influenza preparedness needs to go beyond addressing the viral cause and should also prioritize prevention, diagnosis, prophylaxis, and treatment of secondary bacterial pneumonia as well as stockpiling of antibiotics and bacterial vaccines.^27^

## Risk factors

In direct response to the pandemic, while also more recently, medical officers and researchers set about to define determinants of morbidity and mortality of 1918 pandemic influenza. At Camp Humphreys, a U.S. Army training camp in Virginia, crowding was identified as important risk factor of spread of influenza as its incidence increased with floor space per person in the barracks. One regiment, which had 78·5 ft^2^ per man, had only a 2·5% disease rate, whereas another regiment with 45 ft^2^ per man had an incidence of 26·7%.^30^ Correspondingly, among African American soldiers in an AEF camp in France that were prevented from watching a YMCA performance inside a tent and had to stand outside, none contracted influenza.^4^ Recent analysis of the 1918 pandemic influenza outbreak on New Zealand troop ship Tahiti also implicated crowding, together with inadequate isolation, for >1000 influenza cases and 77 deaths among 1217 persons onboard.^31^

At Camp Lee, another U.S. Army training camp in Virginia, 77% of deaths from influenza and pneumonia were among men with less than three months in service.^4^ Recent analysis of mortality risk factors of 1918 pandemic influenza in the Australian Army revealed that the pneumonia–influenza mortality rate among men who enlisted in 1918 was ∼9 times higher than among the 1917 enlistment cohort and >14 times higher than among the enlistment cohorts of 1916, 1915, and 1914. The protective effect of increased service was hypothesized to reflect increased acquired immunity from previous exposure to influenza viruses and endemic bacterial strains.^32^

By mid-October 1918, it was recognized by the U.S. Army Medical Department that influenza on troop ships could be avoided or minimized by transporting troops who had already had the flu or had been exposed to it.^4^ Based on recently analyzed repeated illness data among seasoned troops in five U.S. Army training camps, the first wave provided 49–94% protection against clinical illness during the second wave and 56–89% protection against death.^12^ Correspondingly, high mortality during the second wave was observed on some warships that had not been affected during the first wave.^33^ Alike, the disproportionately high disease rate onboard New Zealand troop ship Tahiti has also been related to the absence of a first wave in New Zealand before the ship's departure.^31^

**Figure 6:**
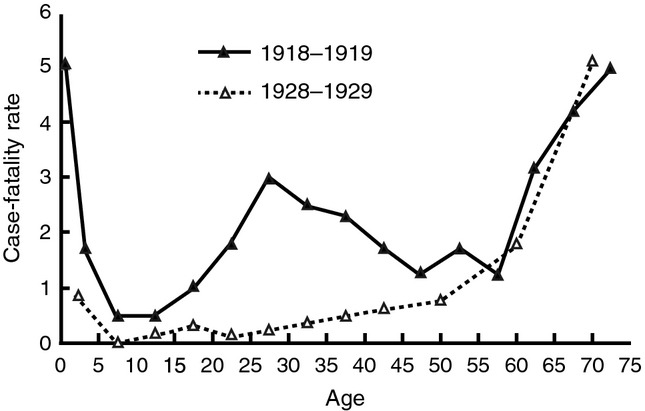
Case-fatality per 100 persons ill with influenza and pneumonia per age group in 1918 (U.S. Public Health Service house-to-house surveys, 8 states) and 1928–1929 (U.S. Public Health Service surveys). The unique W-shaped curve illustrates the high case-fatality rate among infants, young adults, and elderly in 1918 (published in reference 35; publication in the public domain).

A reportedly unique characteristic of 1918 pandemic influenza is the unprecedented mortality rate in persons 20–40 years of age which brought about the so-called W-shaped age-specific mortality curve (highest among infants, young adults, elderly; Figure6).^34^–^39^ Recently, Shanks and Brundage postulated that mortality rates were highest among persons with prior exposures to heterosubtypic influenza strains and limited exposure to other respiratory infectious agents, thereby enhancing immunopathogenic effects upon infection with 1918 pandemic influenza in a context of limited protective antibodies against bacterial strains. In 1918, persons <17 years were less likely to be exposed to influenza A heterosubtypes, while persons >43 years were likely exposed to more respiratory bacterial infections leaving persons 18–43 years of age as the most vulnerable population besides infants and the elderly.^34^

## Influenza produced no heroes

**Figure 7:**
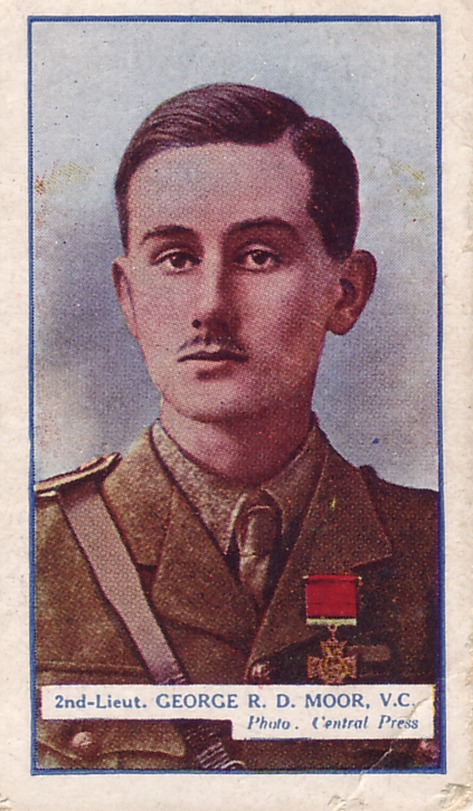
While “influenza produced no heroes”, heroes themselves were not immune to the disease. British Army officer George R.D. Moor, who was awarded the Victoria Cross, the Military Cross, and an additional Bar to the Military Cross, died from 1918 pandemic influenza on November 3, 1918 at the age of 22. He is depicted on a so-called cigarette card commemorating First World War Victoria Cross recipients issued by tobacco company Gallaher Ltd (collection of first author).

In view of the reigning German soldier cult, which heroicized death and sacrifice on the “altar of the fatherland”,^3^ it can be considered far from glorious for a soldier to have died from influenza in a hospital bed behind the front line, removed from trenches, mud, barbed wire, shells, and bullets. Likewise, in the U.S. Army, death in combat was valorized over death by disease. The most glorious was to be killed in action against the enemy, or to die of combat wounds. Only “weaklings” died in bed of a fever.^4^ Yet, while “influenza produced no heroes”,^4^ heroes themselves were not immune to the disease. British Army officer George R.D. Moor, who was awarded the Victoria Cross, the highest British military decoration for valor in the face of the enemy, the Military Cross, a British military decoration in recognition of exemplary gallantry against the enemy on land, and an additional Bar to the Military Cross, died from 1918 pandemic influenza on November 3, 1918 at the age of 22 (Figure7).^40^

**Figure 8:**
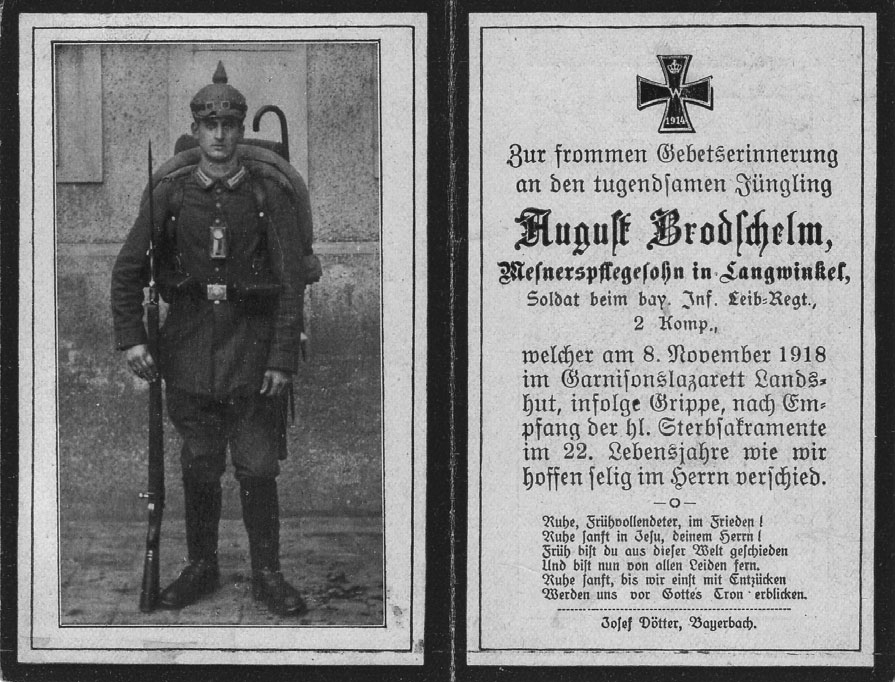
“Sterbebild” (death card) of German private August Brodschelm who died on November 8, 1918 at the age of 22 from “Grippe” (collection of first author).

Even though the German Army recorded 14 000 influenza fatalities,^3^ “Sterbebilder” (death cards) of German soldiers distributed by the next of kin rarely mention influenza as specific cause of death. Rare examples are the death cards of 22-year-old private August Brodschelm who died from “Grippe” on November 8, 1918, and of 24-year-old private Franz X. Bauer who died “den Heldentod fürs Vaterland” (the heroic death for the fatherland) because of “der Grippe” after 48 months of army service on November 19, 1918, eight days after the Armistice (Figure8). More often, influenza as cause of death seems to be referred to on death cards in general terms such as “tödlichen Krankheit” (deadly disease) or “im Felde geholten Krankheit” (disease contracted on the battlefield). In parallel, it has been noted that American WWI cemeteries in France bear the names of great battles like “St. Mihiel American Cemetery” and “Meuse-Argonne American Cemetery”, while none are named after the diseases that contributed to populate them. In the aftermath of the war, one military medical historian even went so far as to state that “in World War I the American Expeditionary Forces suffered no major epidemic problems”, which is illustrative of the dismissal of the influenza epidemic as unimportant or even to ignore it altogether.^4^

## Conclusion

Although 1918 pandemic influenza might have claimed toward 100 000 fatalities among soldiers overall during the conflict and rendered millions ineffective, it remains unclear to this day whether the disease had an impact on the course of WWI. While, for instance, commanding officers complained that the flu was affecting fighting strength, planned offensives had to be delayed, and the morale was further lowered, the effects of 1918 pandemic influenza in purely military terms were probably minimal, even during the second wave. This resulted from the extremely virulent nature of the virus, which came, killed, and moved on. Yet, while 1918 pandemic influenza may have been of little significance militarily, it was a disaster of enormous magnitude from a purely human point of view.^3^

In conclusion, during WWI, 1918 pandemic influenza had a profound impact on both the military apparatus and the individual soldier, but presumably less on the course of the war. Even until this day, virological and bacteriological analysis of preserved archived remains of soldiers that succumbed to 1918 pandemic influenza has important implications for preparedness for future pandemics.
